# Integrated transcriptomic and metabolomic analyses reveal light-quality-dependent regulation of specialized metabolism in *Pseudostellaria heterophylla*

**DOI:** 10.1186/s12864-026-12717-8

**Published:** 2026-03-07

**Authors:** Lin Tang, Yang Yang, Jinghong Jiang, Hua He, Jiwen Liu, Qian Xu, Guoping Shu

**Affiliations:** 1 Guizhou University of Traditional Chinese Medicine, Guiyang, 550025 China; 2 Guizhou Key Laboratory for Germplasm Innovation and Resource-Efficient Utilization of Dao-di Herbs, Guiyang, 550025 China

**Keywords:** Pseudostellaria heterophylla, Light quality, Transcriptome, Metabolome, Specialized metabolism, Phenylpropanoid–flavonoid pathway

## Abstract

*Pseudostellaria heterophylla* is a medicinal and edible plant whose tuberous roots accumulate diverse bioactive metabolites. Light quality is a key environmental signal regulating plant metabolism, but its role in *P. heterophylla* remains unclear. Here, we integrated transcriptomic and widely targeted metabolomic analyses of in vitro–grown tuberous roots exposed to red, blue, and white light. Distinct light-dependent transcriptional and metabolic patterns were detected. Red light mainly enhanced primary metabolism, particularly amino acid-related pathways, while suppressing several specialized metabolites. In contrast, blue light strongly promoted phenylpropanoid- and flavonoid-related metabolites, whereas white light yielded a more balanced metabolic profile. Integrated pathway enrichment and correlation analyses identified the phenylpropanoid and flavonoid network as a central hub of spectral regulation. Overall, these results provide a systems-level view of light-quality-driven metabolic reprogramming in *P. heterophylla* and a mechanistic basis for optimizing LED spectral strategies in tissue culture and controlled cultivation systems.

**Supplementary Information:**

The online version contains supplementary material available at 10.1186/s12864-026-12717-8.

## Introduction

Light is a key environmental signal governing plant growth, development, and specialized metabolism [1, 2]. As the primary driver of photosynthesis and a central regulator of metabolic plasticity, light shapes plant productivity and the biosynthesis of bioactive compounds important for human nutrition and medicine [3, 4]. Besides supplying energy for photosynthesis, light acts as a signaling cue that regulates morphogenesis, circadian rhythms, and specialized metabolite biosynthesis [5, 6]. Light quality, defined by spectral composition, is particularly influential because specific wavelengths are perceived by dedicated photoreceptors, including phytochromes (red/far-red light), cryptochromes and phototropins (blue light), and UVR8 (UV-B light). These photoreceptors initiate signaling cascades that reprogram gene expression and metabolic activity [7, 8]. In medicinal plants, such regulation strongly affects the accumulation of flavonoids, alkaloids, terpenoids, and phenolic acids [9, 10], thereby determining pharmacological efficacy and medicinal value.

Numerous studies have demonstrated that light-mediated signalling modulates specialized metabolism in medicinal species. For instance, blue light stimulates flavonoid biosynthesis in *Scutellaria baicalensis* through MYB and bHLH transcription factors [11], whereas red light enhances anthocyanin accumulation in *Vitis vinifera* via phytochrome-dependent pathways [12]. In *Artemisia annua*, artemisinin (a sesquiterpene antimalarial lactone) biosynthesis is regulated by light through photoreceptor-controlled transcription of terpene synthase genes [13]. Likewise, in *Salvia miltiorrhiza*, light quality controls tanshinone and phenolic acid production through coordinated transcriptional networks [14]. Collectively, these studies indicate that targeted manipulation of light spectra is an effective strategy to improve the yield and quality of medicinal phytochemicals.

*Pseudostellaria heterophylla* (Miq.) Pax, known as “Taizishen”, is a traditional Chinese medicinal herb used as both food and medicine. It exhibits immunomodulatory, anti-fatigue, antioxidant, and anti-inflammatory activities [15, 16], whichare attributed to diverse metabolites, including flavonoids, phenolic acids, alkaloids, amino acids, and cyclic peptides. Among these, flavonoids and phenolic acids are major contributors to antioxidant and anti-inflammatory effects [15, 16], whereas alkaloids and amino acid derivatives are implicated in energy metabolism and immune regulation [17, 18]. However, the biosynthetic pathways and regulatory mechanisms controlling these metabolites remain poorly defined, and the environmental factors governing their accumulation are poorly characterized.

The tuberous root of *P. heterophylla*, the primary medicinal organ, functions both as a storage tissue and as an active site of secondary metabolite biosynthesis [19]. In current production, this species is commonly cultivated under shaded or forested conditions with complex and dynamic light environments and is increasingly introduced into greenhouse and plant factory systems for seed tuber production and quality control. Although LED lighting enables precise spectral regulation in these protected systems, practical light regimes that concurrently optimize tuber yield and medicinal quality remain undefined. Moreover, despite extensive studies in other medicinal and horticultural crops, the role of light quality in regulating specialized metabolism in *P. heterophylla* is poorly understood. Understanding how light quality modulates the accumulation of key bioactive components is therefore critical for both mechanistic insight and cultivation optimization.

High-throughput omics approaches offer effective means to elucidate light-mediated metabolic regulation. Transcriptomics enables genome-wide analysis of gene expression responses to light conditions, while metabolomics quantitatively captures metabolite dynamics [20, 21]. Integrated transcriptomic and metabolomic analyses can reveal gene–metabolite associations, identify regulatory nodes, and construct mechanistic models of light-controlled biosynthetic pathways [22]. Such strategies have yielded important insights into medicinal plants including *Camellia sinensis*, *Glycyrrhiza uralensis*, and *Panax ginseng*, highlighting transcriptional control of specialized metabolism and its environmental sensitivity [23–25].

This study subjected in vitro plantlets of *P. heterophylla* to three light-quality regimes (red, blue, and white light) to assess how spectral composition influences specialized metabolism. Microtubers induced under each treatment were subjected to integrated transcriptomic and metabolomic analyses. The objectives were to: (i) characterize global transcriptomic and metabolomic responses to different light qualities; (ii) identify key metabolic pathways and candidate genes involved in light-regulated specialized metabolism, with particular emphasis on the phenylpropanoid–flavonoid pathway; and (iii) infer potential regulatory mechanisms by integrating gene expression with metabolite accumulation patterns. Collectively, these results advance understanding of light-quality-dependent metabolic regulation in *P. heterophylla* and provide a theoretical basis for optimizing spectral conditions in LED-based nursery production, microtuber induction, and protected cultivation to enhance medicinal quality. Unlike previous studies that primarily examined aerial tissues of other species, this study focuses on in vitro-induced microtubers of *P. heterophylla* and delineates spectrum-dependent trade-offs between primary metabolism and specialized metabolite accumulation, offering practical guidance for LED recipes in seed-tuber production.

## Materials and methods

### Plant materials

*P. heterophylla* (cultivar “Shitai 1”) seeds were obtained from the experimental base of Guizhou University of Traditional Chinese Medicine (Guiyang, China). Uniform, well-developed seeds were selected and soaked in distilled water for 20 h. The outer seed coat was gently removed using sterile gauze, and seeds were rinsed under running water for 30 min to remove surface impurities. Under aseptic conditions in a laminar-flow cabinet, seeds were surface-sterilized by immersion in 75% (v/v) ethanol for 1 min, rinsed thrice with sterile distilled water, treated with 2.5–3.0% (v/v) sodium hypochlorite for 6 min, and rinsed five times with sterile distilled water. Intact embryos were excised and inoculated on Murashige and Skoog (MS) medium for germination under sterile conditions (16 h light/8 h dark photoperiod, 25 ± 2 °C, 75% relative humidity). After 15 days, aseptic seedlings were obtained. Stem segments from these seedlings were excised and subcultured on fresh MS medium to produce tissue-cultured plantlets for subsequent experiments. Under in vitro conditions, the underground parts of *P. heterophylla* plantlets enlarged, forming microtuber-like structures morphologically similar to natural tuberous roots. These microtubers were collected for transcriptomic and metabolomic analyses.

### Light-quality treatments

*P. heterophylla* plantlets sub-cultured on MS medium for 40 days were exposed to red (620 nm), blue (420 nm), or white LED light for 30 days. White light served as the broad-spectrum reference, reflecting standard tissue-culture illumination. Pairwise comparisons (Red_vs._White, Blue_vs._White, Blue_vs._Red) were used to assess wavelength-specific effects. For the white-light treatment, the measured spectrum showed an RGB composition of R 43.2%, G 37.6%, B 19.2%, with chromaticity coordinates x = 0.3671, y = 0.3516 and a correlated color temperature of 4213 K (dominant wavelength 585.7 nm, peak wavelength 662.4 nm, FWHM 25.3 nm) (Figure S1). Cultures were maintained in an artificial chamber under a 16 h light/8 h dark photoperiod, 25 ± 2 °C, 75% relative humidity, and a photosynthetic photon flux density (PPFD) of 200 µmol·m⁻²·s⁻¹. LED lamps were positioned ~ 50 cm above the culture vessels, and light intensity was regularly calibrated using a digital light meter (LX1330E). Three independent bottles per light condition served as biological replicates, each containing separate plantlets. Tuberous root (Microtubers) were harvested from each bottle and processed individually for RNA-seq and metabolomic analyses.

### RNA extraction and sequencing

Total RNA was extracted from P. heterophylla tuberous roots using the Total Plant RNA Kit (Tiangen Biotech Co., Ltd., China) following the manufacturer’s protocol. RNA integrity and concentration were assessed with an Agilent 2100 Bioanalyzer (Agilent Technologies, USA). Library construction and paired-end sequencing (2 × 150 bp) were performed on the Illumina NovaSeq 6000 platform by Wuhan Metware Biotechnology Co., Ltd. (Wuhan, China) using three biological replicates per treatment.

### Transcriptome analysis

Raw reads were processed with fastp (v0.23.2) to remove adapters and low-quality sequences. Clean reads were de novo assembled using Trinity (v2.15.1), and redundancy was reduced with Corset (v1.09) to generate transcript clusters. Coding sequences (CDSs) were predicted using TransDecoder (v5.3.0). Functional annotation of unigenes was performed with DIAMOND BLASTx (v2.0.9) against NR, Swiss-Prot, Pfam, KOG/COG, GO, and KEGG databases. Gene Ontology (GO) terms were classified into biological process, cellular component, and molecular function categories, while KEGG annotation assigned genes to metabolic and signaling pathways. Gene expression levels were estimated with RSEM (v1.3.1) after alignment using Bowtie2 (v2.4.5) and normalized as FPKM. Differentially expressed genes (DEGs) between light treatments were identified using DESeq2 (v1.22.2) with thresholds of VIP ≥ 1, Fold_Change ≥ 2 or Fold_Change ≤ 0.5, and FDR < 0.05. Functional enrichment of DEGs was performed using clusterProfiler (v4.2.1), considering GO terms and KEGG pathways with q-value < 0.05 as significantly enriched. Visualization (bubble plots, bar plots, heatmaps) and statistical analyses (PCA, sample correlation, hierarchical clustering) were conducted using R and the Metware Cloud platform. BUSCO was run using BUSCO v5.4.3 with the eukaryota_odb10 dataset (comprising 255 conserved single-copy orthologs specific to eukaryotes) to assess the completeness of the de novo transcriptome assembly.

### Sample preparation and metabolite extraction

Tuberous root (microtuber) tissues of *P. heterophylla* were collected after 30 days of exposure to different light treatments. Samples were immediately frozen in liquid nitrogen and stored at -80 °C until use. The frozen materials were vacuum freeze-dried for 63 h using a lyophilizer (Scientz-100 F) and then ground into fine powder with a grinder (MM 400, Retsch, Germany) at 30 Hz for 1.5 min. A 30 mg aliquot of powder was extracted with 1.5 mL of pre-cooled (-20 °C) 70% methanol containing internal standards. The internal standard working solution (250 µg mL⁻¹) was prepared by dissolving 1 mg of reference compound in 1 mL of 70% methanol to obtain a 1000 µg mL⁻¹ stock solution, which was then diluted accordingly. During extraction, samples were vortexed for 30 s every 30 min, six times in total. Extracts were centrifuged at 12,000 rpm for 3 min, and the supernatant was filtered through a 0.22 μm membrane before UPLC-MS/MS analysis.

### UPLC-MS/MS analysis and data processing

Metabolite profiling was performed using an ExionLC™ AD UPLC system coupled with a triple quadrupole mass spectrometer (MS/MS). Chromatographic separation was achieved on an Agilent SB-C18 column (1.8 μm, 2.1 mm × 100 mm) using mobile phases of (A) 0.1% formic acid in water and (B) 0.1% formic acid in acetonitrile. The gradient elution program was: 0–9.00 min, 5–95% B; 9.00–10.00 min, hold at 95% B; 10.00–11.10 min, decrease to 5% B; and 11.10–14.00 min, re-equilibration at 5% B. The flow rate was 0.35 mL min⁻¹, column temperature 40 °C, and injection volume 2 µL.

Mass spectrometry was conducted using an electrospray ionization (ESI) source in both positive and negative ion modes. Source temperature was 500 °C, with spray voltages of + 5500 V (positive) and − 4500 V (negative). Gas settings were: curtain gas = 25 psi, ion source gas 1 = 50 psi, ion source gas 2 = 60 psi. Multiple reaction monitoring (MRM) mode was used for quantification, with nitrogen as the collision gas. MRM parameters (DP, CE) were optimized for each metabolite. Metabolites were identified by matching MS/MS spectra against the Metware Database (MWDB, Wuhan Metware Biotechnology Co., Ltd.) and quantified using MRM peak areas. Peak areas were normalized across samples following Fu et al. [26].

Data were processed in Microsoft Excel. PCA, hierarchical clustering, volcano plots, and heatmaps were generated using the Metware Cloud Platform and TBtools II. Metabolites with VIP ≥ 1, Fold_Change ≥ 2 or Fold_Change ≤ 0.5, and FDR < 0.05 were considered differentially accumulated metabolites (DAMs). Functional annotation and pathway enrichment analyses were performed using KEGG and MetMap databases. The confidence level criteria for metabolite annotation with three graded levels. Level 1: The matching score of MS/MS full fragment ions and retention time (RT) between the metabolites in samples and the database is ≥ 0.7; Level 2: The matching score of MS/MS full fragment ions and RT between the metabolites in samples and the database ranges from 0.5 to 0.7; Level 3: The precursor ion (Q1), product ion (Q3), RT, declustering potential (DP) and collision energy (CE) of the metabolites in samples are completely consistent with the database information.

### Quantitative reverse transcription PCR

Quantitative reverse transcription PCR (qRT-PCR) was conducted with gene-specific primers (Table S1) under the following conditions: 95 °C for 3 min, followed by 44 cycles of 95 °C for 5 s and 60 °C for 20 s. Actin served as the internal reference. Relative expression was calculated using the 2^−ΔΔCt^ method. Statistical differences among treatments were evaluated by one-way ANOVA followed by Tukey’s multiple comparison test (*P* < 0.05).

## Results

### Transcriptome profiling of P. heterophylla under different light-quality treatments

**Fig. 1:**
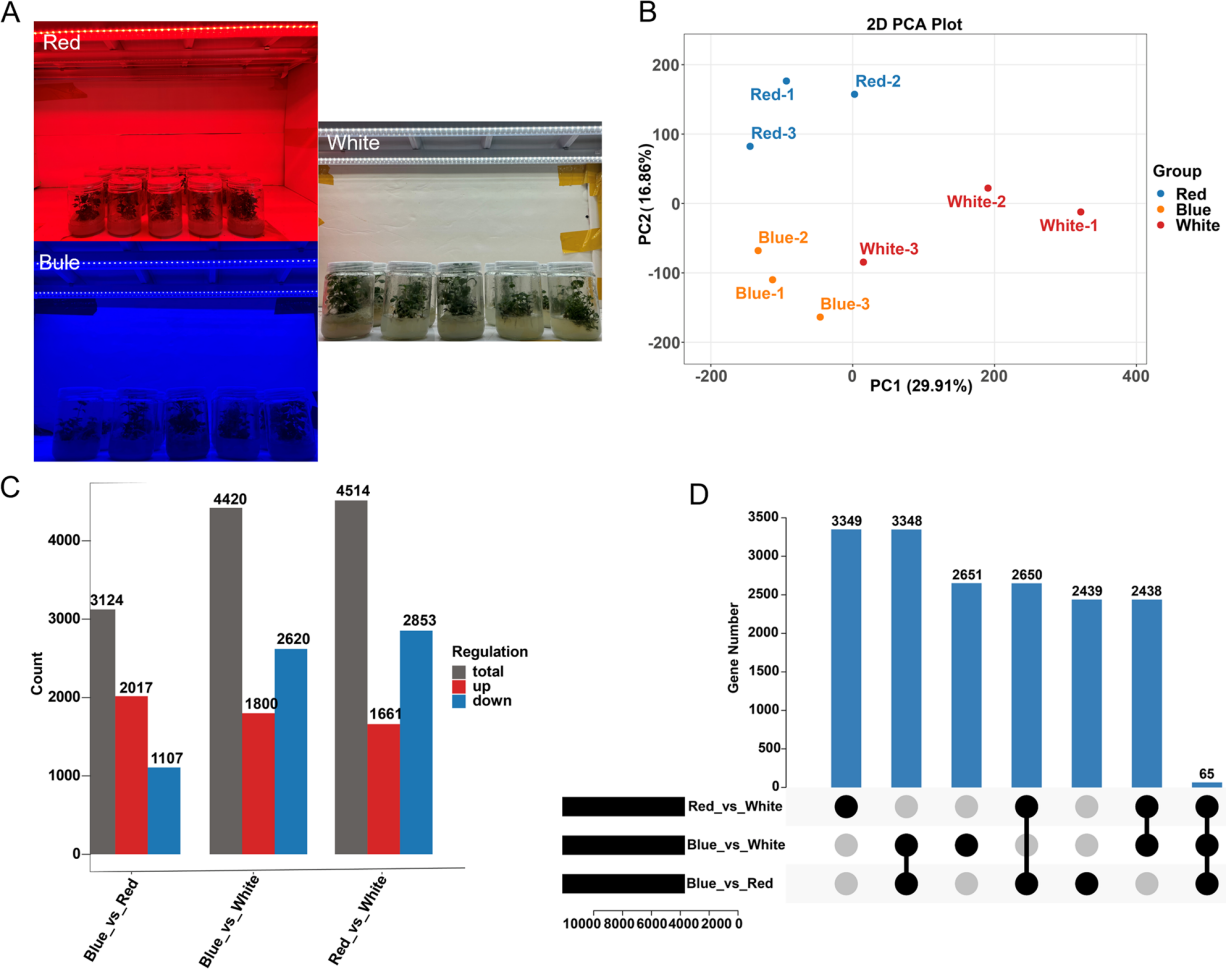
Transcriptomic analysis of *P. heterophylla* samples under different light-quality treatments. (**A**) Photographs of *P. heterophylla* seedlings under red, blue, and white light treatments. (**B**) Principal component analysis (PCA) of transcriptomic data showing tight clustering of biological replicates within each treatment and clear separation among the three light-quality groups. (**C**) Numbers of significantly up-regulated and down-regulated DEGs identified in three pairwise comparisons (VIP ≥ 1, Fold_Change ≥ 2 or Fold_Change ≤ 0.5, and FDR < 0.05). (**D**) UpSet plot illustrating the overlap and specific DEGs among the three pairwise comparisons (Red_vs._White, Blue_vs._Red, and Blue_vs._White)

To investigate how light quality regulates tuberous-root metabolism in *P. heterophylla*, we performed RNA-seq on roots exposed to red, blue, and white light (Fig. 1A). Using the Illumina platform, 4.61 × 10⁸ raw reads (74.1 Gb) were generated. After quality filtering, 70.3 Gb of clean data were retained, with 6.35–8.84 Gb per sample. The sequencing error rate was < 0.01%, Q20 and Q30 values were 99.4–99.5% and 97.2–97.5%, respectively, and GC content ranged from 43.6% to 44.1%, indicating high-quality transcriptome data (Table S2). Trinity de novo assembly produced 142,844 transcripts (mean length 1,379 bp; N50, 2,281 bp; N90, 616 bp). After redundancy removal using Corset, 84,963 unigenes were retained (mean length 1,716 bp; N50, 2,474 bp; N90, 800 bp), representing a 40.5% reduction in redundancy (Table S3). Functional annotation assigned 72.11% of unigenes (61,271) to at least one public database, with annotation rates of 70.02% (Nr), 51.50% (Swiss-Prot), 53.57% (KEGG), 60.85% (GO), and 53.32% (Pfam) (Table S4). BUSCO analysis indicated 100% completeness (C:255; 45 single-copy and 210 duplicated; F:0; M:0; *n* = 255), confirming the integrity of the assembly (Figure S2).

Principal component analysis (PCA) showed tight clustering of biological replicates within each treatment and clear separation among red, blue, and white light conditions along PC1 (29.91%) and PC2 (16.86%), indicating strong light-quality-dependent transcriptional differences (Fig. 1B). We identified 23,068 DEGs across all pairwise comparisons (Fig. 1C). Specifically, 3,124 DEGs were detected in Blue_vs._Red (2,017 up- and 1,107 down-regulated), 4,420 in Blue_vs._White (1,800 up- and 2,620 down-regulated), and 4,514 in Red_vs._White (1,661 up- and 2,853 down-regulated). UpSet analysis revealed limited overlap among comparisons, with only 65 DEGs shared by all three, whereas most DEGs were comparison-specific (Fig. 1D). These results indicate that distinct gene sets are activated under different light qualities, supporting light-specific metabolic regulation in *P. heterophylla*.

To connect light perception with transcriptional regulation, we analyzed key components of the COP1/SPA-centered light signaling pathway. Multiple *COP1* and *SPA* homologs exhibited clear light-quality-dependent expression patterns across replicates (Figure S3). Additionally, several genes involved in amino acid metabolism (e.g., *trpB*, *asnB*, and *TAT*) also showed consistent differential expression among treatments (Figure S4).

### Clustering analysis of light-responsive gene expression patterns in P. heterophylla

**Fig. 2:**
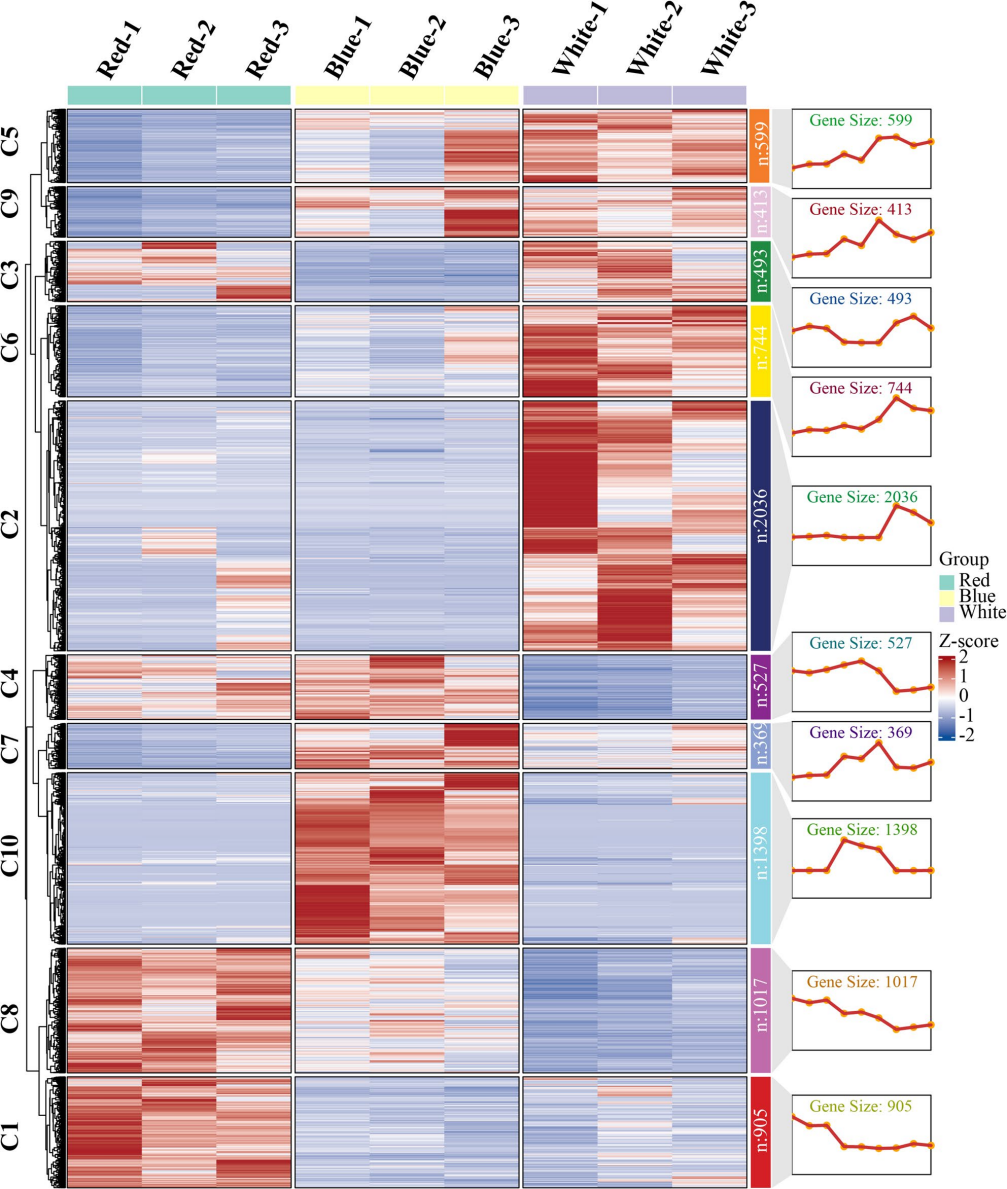
Clustering analysis of DEGs in *P. heterophylla* under different light-quality treatments. Based on expression trends among samples treated with red, blue, and white light, all DEGs were classified into ten expression pattern clusters (C1-C10). The line charts on the right show the average expression trends of each cluster, and the color scale represents the normalized expression level (Z-score)

To further characterize transcriptional responses of *P. heterophylla* to different light qualities, 8,501 DEGs with high expression variability were clustered into ten groups (C1–C10) based on their expression patterns across nine samples (Fig. 2). Each cluster exhibited a distinct response to red, blue, and white light. Clusters C1 (905 genes) and C8 (1,017 genes) were strongly induced by red light but showed marked repression under blue and, especially, white light. In contrast, cluster C10 (1,398 genes) was specifically up-regulated by blue light, with consistently high expression across all blue-light replicates and low expression under red and white light. Cluster C7 (369 genes) also displayed a blue-responsive pattern, with peak expression in Blue-2 and Blue-3 and low expression in most red- and white-light samples.

Cluster C4 (527 genes) showed preferential expression under red light and partially under Blue-1, followed by a pronounced decrease in Blue-2/Blue-3 and all white-light samples. By contrast, clusters C2 (2,036 genes) and C6 (744 genes) were strongly induced under white light and repressed under red and blue light, indicating a gene set specifically responsive to broad-spectrum illumination. Finally, clusters C3 (493 genes), C9 (413 genes), and C5 (599 genes) exhibited mixed response patterns along the red–blue–white gradient. Cluster C3 showed high expression under red and white light but repression under blue light, whereas clusters C9 and C5 displayed a gradual increase in expression from red to blue and white light, with the highest levels generally observed in Blue-2/Blue-3 and white-light samples. Overall, these clustering results reveal multiple light-quality-dependent transcriptional modules in *P. heterophylla*, suggesting that genes within each cluster participate in distinct light-signaling pathways and associated metabolic processes.

### KEGG enrichment highlights light-quality-dependent primary and secondary metabolic pathways

**Fig. 3:**
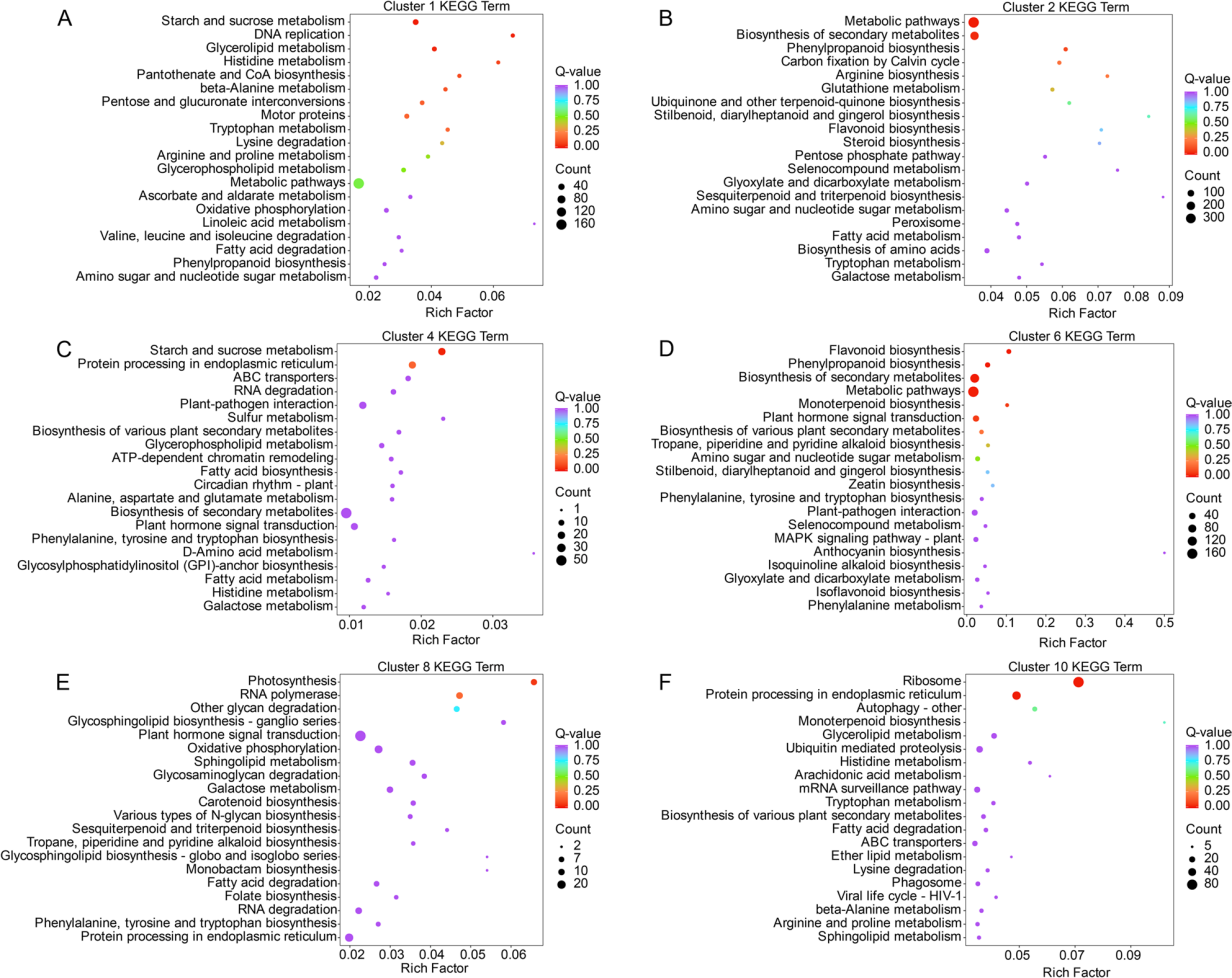
KEGG enrichment analysis of DEGs from six representative light-responsive co-expression clusters in *P. heterophylla*. (**A**-**F**) Top 20 enriched KEGG pathways (q < 0.05) for clusters C1, C2, C4, C6, C8 and C10, respectively. The x-axis indicates the Rich factor, bubble size represents the number of genes in each term, and bubble colour reflects the q-value

To associate co-expression clusters with biological functions, KEGG enrichment analysis was performed for six representative clusters (C1, C2, C4, C6, C8, and C10) exhibiting clear light-quality preferences (Fig. 3). Enriched pathways were largely cluster-specific, indicating that distinct gene sets mediate responses to red, blue, and white light.

The red-light-induced clusters C1 and C8 were mainly enriched in primary metabolic pathways, including “Starch and sucrose metabolism”, “Amino sugar and nucleotide sugar metabolism”, and several amino-acid-related pathways such as “Valine, leucine and isoleucine degradation” and “Tryptophan metabolism”, together with energy- and redox-associated pathways such as “Oxidative phosphorylation” and “Ascorbate and aldarate metabolism” (Fig. 3A, E). Cluster C8 was additionally enriched in “Photosynthesis” and “glycan/lipid metabolism”. Together, these results suggest that red light predominantly reprograms central carbon and nitrogen metabolism and associated energy processes, thereby supplying metabolic precursors that may support downstream specialized metabolism.

In contrast, the clusters preferentially expressed under white light (C2 and C6) were strongly enriched in specialized metabolism and defence pathways. C2 was enriched in broad categories such as “Metabolic pathways” and “Biosynthesis of secondary metabolites”, with pronounced enrichment of “Phenylpropanoid biosynthesis” and “Flavonoid biosynthesis” (Fig. 3B). C6 shared these terms and additionally showed enrichment in “Biosynthesis of various plant secondary metabolites”, “Monoterpenoid biosynthesis”, “Tropane, piperidine and pyridine alkaloid biosynthesis”, and “Anthocyanin biosynthesis”, as well as signaling and defence pathways including “Plant-pathogen interaction” and “MAPK signaling pathway-plant” (Fig. 3D). Together, these patterns indicate that white light activates an extensive phenylpropanoid–flavonoid network alongside multiple stress- and hormone-related signalling routes.

Cluster C4, which showed higher expression under red light and in one blue-light replicate but lower expression in other blue- and white-light samples, was enriched in “Protein processing in endoplasmic reticulum”, “Plant–pathogen interaction”, “Plant hormone signal transduction”, “Circadian rhythm–plant”, and several amino acid and lipid metabolism pathways (Fig. 3C). This suggests that C4 functions as a regulatory module linking light signalling, hormonal control, and protein quality regulation.

The blue-light-specific cluster C10 was mainly enriched in protein turnover pathways, including “Ribosome”, “Protein processing in endoplasmic reticulum”, “Ubiquitin mediated proteolysis”, and “Autophagy–other”, together with “Monoterpenoid biosynthesis” and “Biosynthesis of various plant secondary metabolites” (Fig. 3F). These results indicate that blue light promotes active protein synthesis and degradation while selectively modulating terpenoid and other secondary metabolic pathways.

Overall, the KEGG enrichment profiles support a model in which red light primarily regulates primary metabolism and energy supply, whereas white and blue light preferentially activate phenylpropanoid–flavonoid and other secondary metabolic pathways, consistent with the metabolomic trends described below.

### Metabolomic characteristics under different light qualities

To link transcriptional changes with metabolic outcomes, a widely targeted metabolomic analysis was conducted on *P. heterophylla* tuberous roots grown under red, blue, and white light. A total of 2,139 metabolites were detected and classified into 12 major groups, with flavonoids (437), amino acids and derivatives (315), and alkaloids (246) representing the most abundant secondary or nitrogen-containing classes (Table S5). The high abundance of flavonoids, phenolic acids, terpenoids, and lignans/coumarins highlights the prominence of phenylpropanoid- and terpenoid-derived metabolism in this species.

**Fig. 4:**
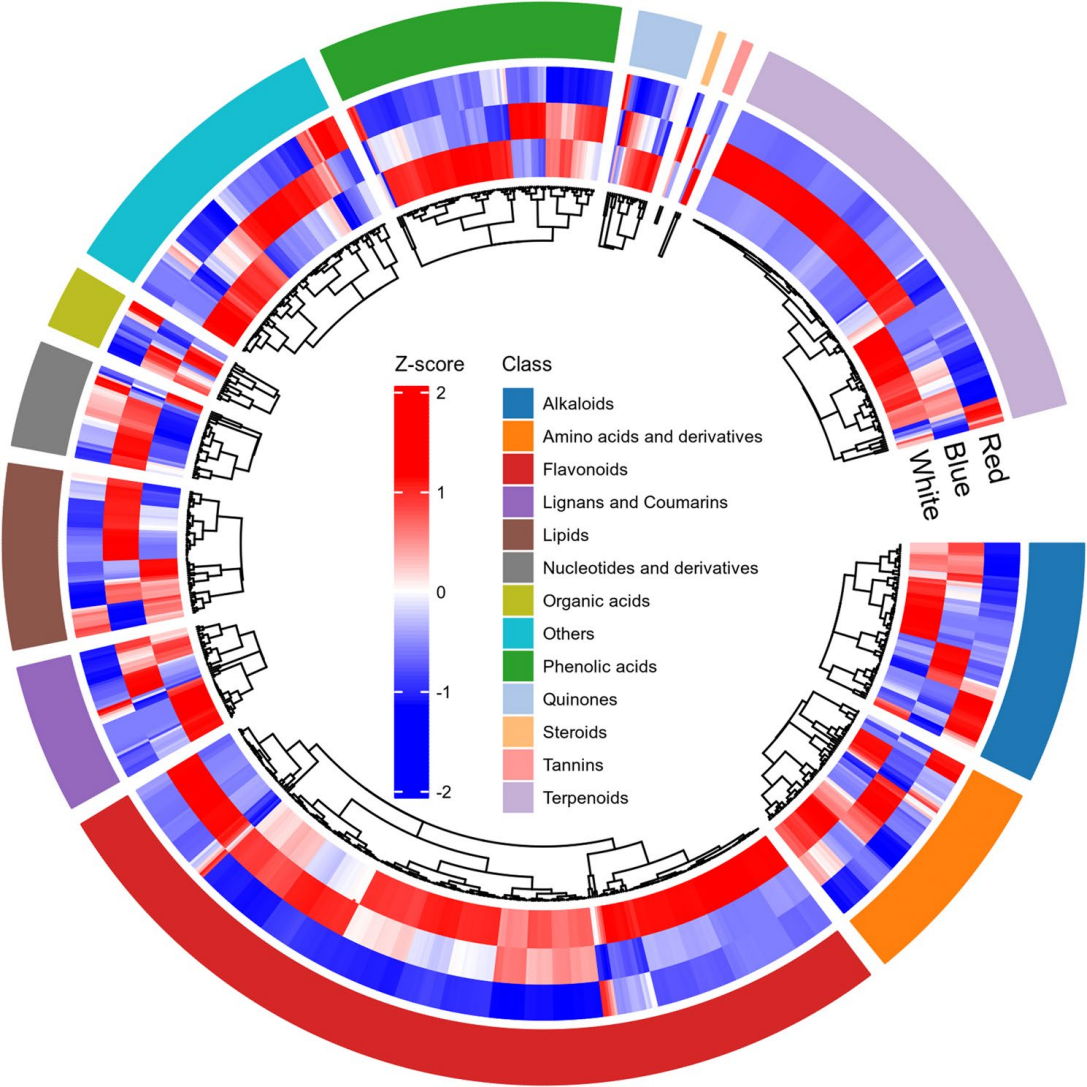
Global metabolite profiles of *P. heterophylla* tuberous roots under different light-quality treatments. Circular heatmap of 2,139 identified metabolites classified into 12 major categories. Each row corresponds to one metabolite, and colours represent Z-score-normalized relative abundances (red, high; blue, low). The outer ring indicates the three light treatments (red, blue and white). Distinct patterns highlight light-quality-specific regulation of primary and secondary metabolites

Circular heatmap analysis revealed clear light-quality-dependent accumulation patterns (Fig. 4). Red light increased most amino acids and their derivatives, along with a subset of lipids, indicating enhanced nitrogen assimilation and central metabolism. Blue light markedly increased flavonoids, lignans, and coumarins while reducing many lipid species, suggesting carbon reallocation from storage lipids to aromatic secondary metabolites. White light produced a more balanced profile, with generally higher levels of organic acids, phenolic acids, and terpenoids than red light, indicating coordinated activation of both primary and specialized metabolism under broadband illumination.

### Differential metabolite profiles under different light-quality treatments

**Fig. 5:**
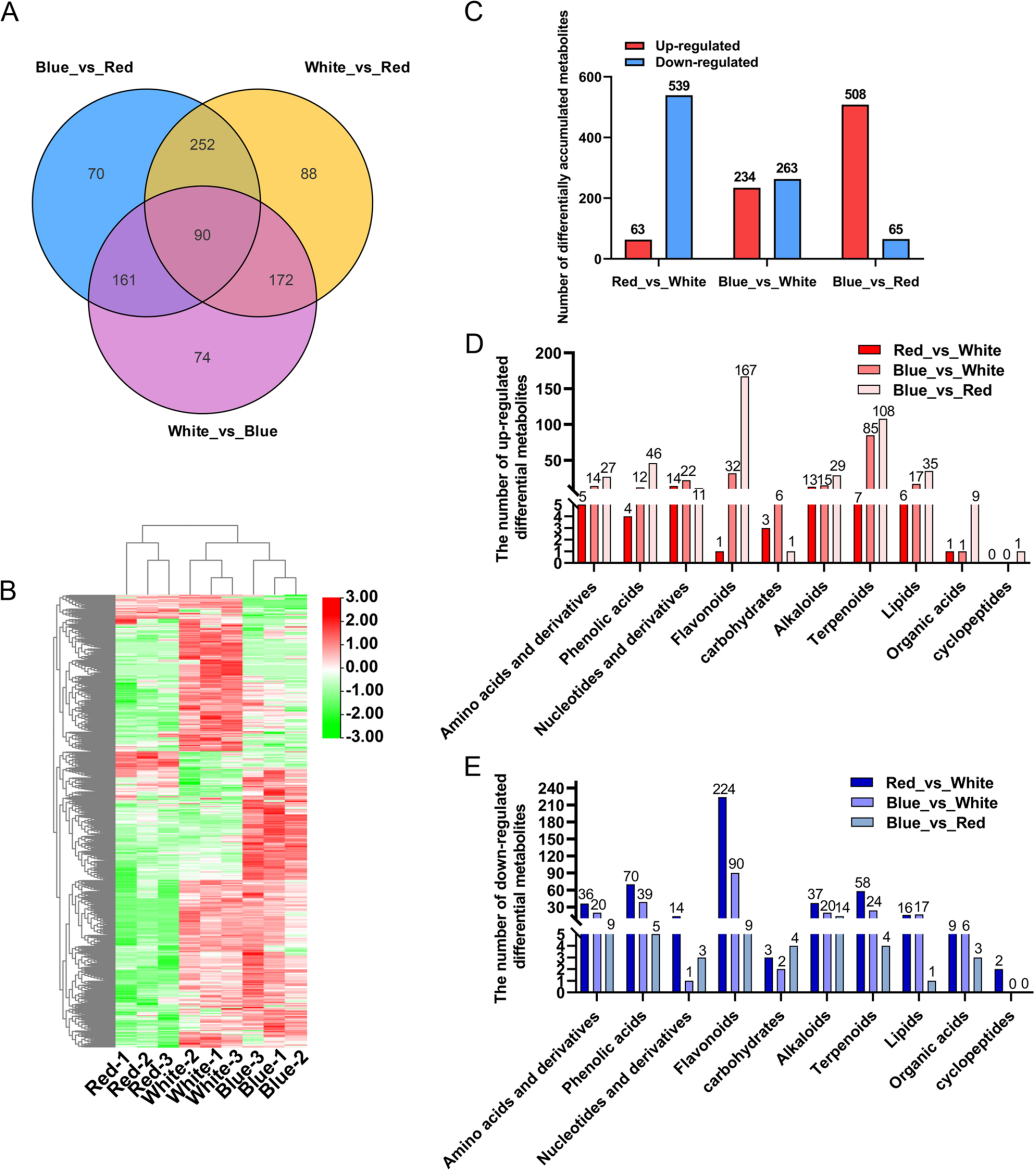
Differentially accumulated metabolites (DAMs) in *P. heterophylla* under red, blue and white light treatments. (**A**) Venn diagram showing the overlap of DAMs among Red_vs._White, Blue_vs._White and Blue_vs._Red comparisons. (**B**) Hierarchical clustering heatmap of all DAMs across three light treatments (three biological replicates each). (**C**) Numbers of upregulated (red) and downregulated (blue) DAMs in each comparison. (**D**-**E**) Classification of upregulated (D) and downregulated (E) DAMs according to metabolic category. DAMs were defined as metabolites with fold change > 2 and *P* < 0.05

To identify metabolites most strongly associated with light spectrum-specific responses, differentially accumulated metabolites (DAMs) were defined using a VIP ≥ 1, Fold_Change ≥ 2 or Fold_Change ≤ 0.5, and FDR < 0.05. Across three pairwise comparisons, 908 DAMs were identified (Table S5). Only 90 DAMs were shared among all comparisons, whereas each contrast contained many unique DAMs (Fig. 5A), indicating strong treatment-specific metabolic reprogramming. Hierarchical clustering clearly separated blue-light samples from red- and white-light samples, which clustered more closely together (Fig. 5B), consistent with the transcriptomic PCA results.

In total, 602, 497, and 573 DAMs were detected in Red_vs._White, Blue_vs._White, and Blue_vs._Red, respectively (Fig. 5C). In Red_vs._White, most DAMs (539/602) were down/regulated under red light. These decreases mainly involved flavonoids, phenolic acids, terpenoids, and alkaloids, whereas only a small number of phenolic acids, lipids, and organic acids were modestly increased. This pattern indicates that red light broadly suppresses specialized metabolite accumulation relative to white light.

In contrast, blue light strongly promoted metabolite accumulation. In Blue_vs._Red, 508 of 573 DAMs were more abundant under blue light, with flavonoids (167 compounds) and terpenoids (108) as the dominant classes, followed by lipids, phenolic acids, alkaloids, and amino acid derivatives (Fig. 5D). Several bioactive metabolites, including apigenin-7-O-glucoside and oleanolic acid, increased more than fivefold relative to red light. Compared with white light, blue light also increased flavonoids and phenolic acids, although the magnitude of change was smaller (Fig. 5D, E). Overall, these results demonstrate that blue light enhances the accumulation of phenylpropanoid- and terpenoid-derived metabolites, whereas red light exerts a generally suppressive effect.

### Integrated transcriptomic and metabolomic analysis highlights coordinated regulation of phenylpropanoid-derived metabolism

**Fig. 6:**
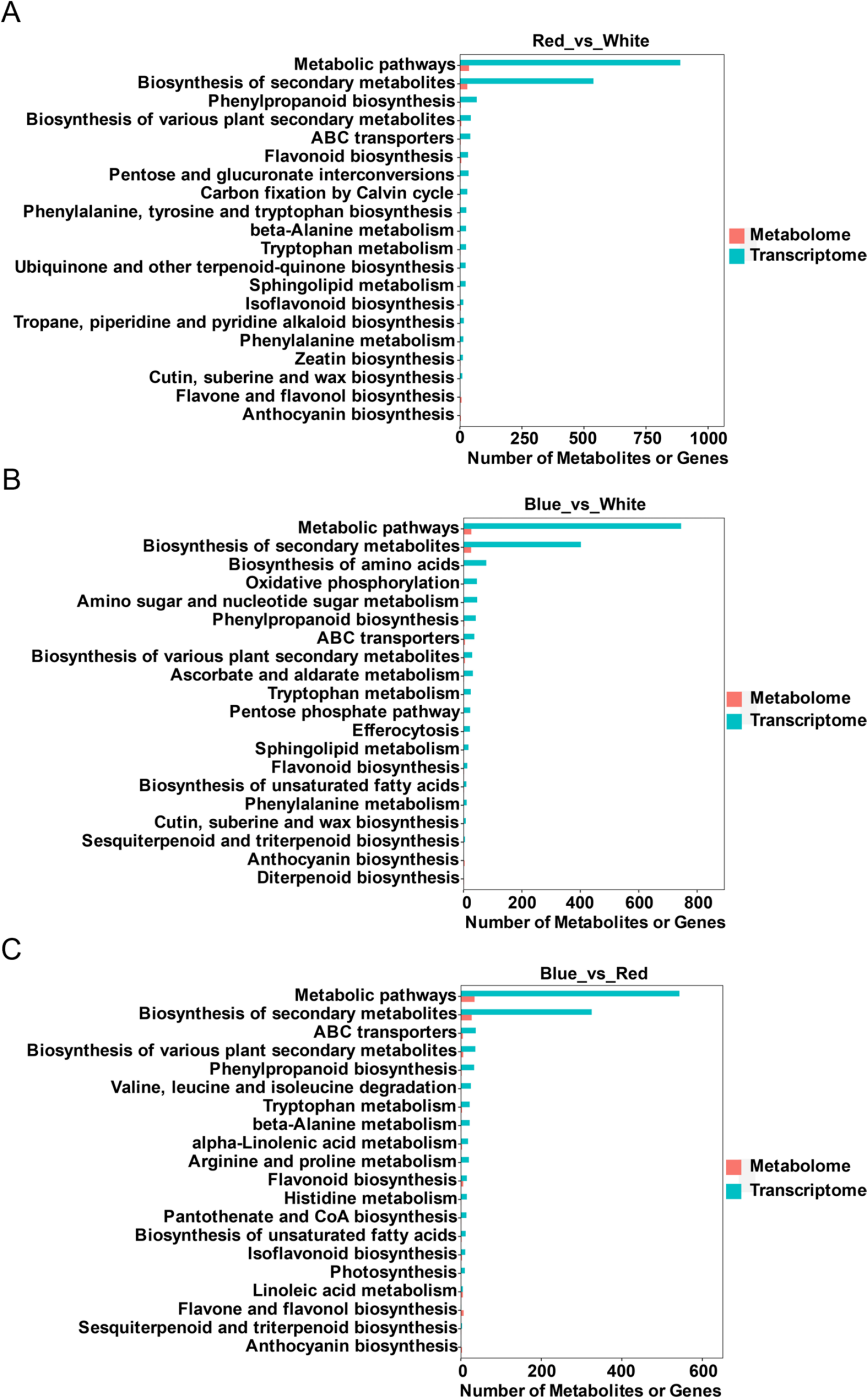
Integrated KEGG enrichment of DEGs and DAMs in *P. heterophylla* under different light-quality treatments. **A**-**C** Overlapping KEGG pathways enriched in both DEGs and DAMs for Red_vs._White (**A**), Blue_vs._White (**B**) and Blue_vs._Red (**C**). Bubble plots display the top shared pathways; bubble size indicates the number of DEGs/DAMs mapped to each term, and colour represents the q-value

To link transcriptional regulation with metabolic changes, KEGG enrichment analyses of DEGs and DAMs were jointly performed for each comparison (Fig. 6). In all contrasts, “Metabolic pathways” and “Biosynthesis of secondary metabolites” were significantly enriched in both datasets. Notably, the most consistent and biologically meaningful overlap involved phenylpropanoid and flavonoid biosynthesis pathways.

In Red_vs._White, the DEGs and DAMs were co-enriched in “Phenylpropanoid biosynthesis”, “Flavonoid biosynthesis”, and several amino acid- and carbon-related pathways, including “Phenylalanine, tyrosine and tryptophan biosynthesis” and “Pentose and glucuronate interconversions” (Fig. 6A). The predominance of downregulated DAMs, together with reduced expression of many flavonoid-related genes under red light, indicates that phenylpropanoid flux into specialized branches is lower than under white light.

In Blue_vs._White, DEGs and DAMs again showed overlapping enrichment in “Phenylpropanoid biosynthesis” and “Flavonoid biosynthesis”, together with “Biosynthesis of unsaturated fatty acids”, “Biosynthesis of amino acids”, “Oxidative phosphorylation” and “Amino sugar and nucleotide sugar metabolism” (Fig. 6B). This pattern suggests that blue and white light jointly regulate energy metabolism and precursor supply while promoting phenylpropanoid-derived specialized metabolism.

In Blue_vs._Red, integrated analysis revealed strong co-enrichment of phenylpropanoid and flavonoid biosynthesis, “Biosynthesis of various plant secondary metabolites”, and several amino-acid and fatty-acid pathways, notably “Valine, leucine and isoleucine biosynthesis”, “Linoleic acid metabolism” and “α-Linolenic acid metabolism” (Fig. 6C). These results indicate that, compared with red light, blue light enhances both precursor metabolism and downstream aromatic and terpenoid biosynthetic branches.

Collectively, these analyses identify the phenylpropanoid–flavonoid pathway as a central target of light-quality regulation, acting as a metabolic hub that integrates changes in primary metabolism with secondary metabolite accumulation.

### Coordinated regulation of the phenylpropanoid-flavonoid network under different light qualities

**Fig. 7:**
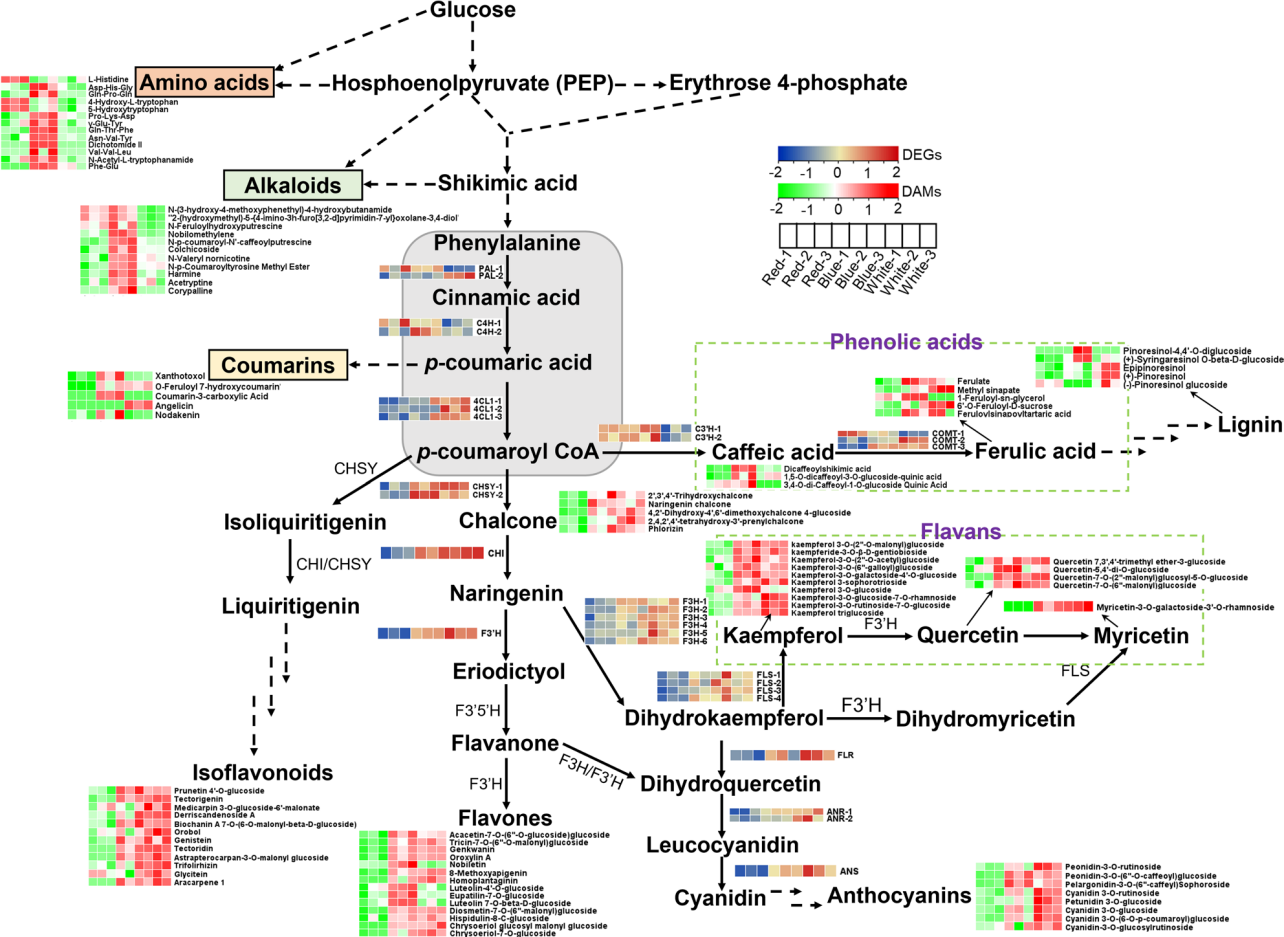
Integrated phenylpropanoid–flavonoid metabolic network regulated by light quality in *P. heterophylla*. Schematic representation of key enzymatic steps from phenylalanine to flavonoids, phenolic acids, lignans and coumarins. Heatmaps next to enzyme names show relative expression levels of corresponding DEGs under red, blue and white light treatments. Heatmaps next to metabolite names indicate relative abundances of DAMs. Solid arrows represent single enzymatic steps, while dashed arrows indicate multi-step conversions. Full names of enzyme abbreviations and compound names are provided in the Abbreviations section (Table S6)

To resolve pathway-level regulation in greater detail, DEGs and DAMs were mapped onto a curated phenylpropanoid–flavonoid pathway model (Fig. 7). This model includes core phenylpropanoid enzymes (PAL, C4H, 4CL), chalcone and flavanone biosynthetic enzymes (CHS, CHI, F3H), hydroxylases and O-methyltransferases (F3′H, F3′5′H, C3′H, COMT), and branch-specific enzymes involved in flavonol, anthocyanin, and proanthocyanidin biosynthesis (FLS, DFR, ANS, ANR).

Among the 31 structural genes analyzed, 26 showed higher expression under blue and/or white light than under red light. The upstream genes *PAL*, *C4H*, and *4CL* were induced by both red and blue light, indicating that pathway entry steps broadly respond to spectral variation. In contrast, many downstream flavonoid-branch genes, including *CHS*, *CHI*, *F3H*, *F3′H*, and *FLS*, were preferentially upregulated under blue and white light, consistent with the enhanced accumulation of multiple flavonoid subclasses in these treatments.

At the metabolite level, 34 of the 52 identified flavonoids accumulated to higher levels under blue and/or white light than under red light. These DAMs spanned several subclasses, including chalcones (e.g., 2′,3′,4′-trihydroxychalcone and naringenin chalcone), kaempferol- and quercetin-derived flavonols, tricin and luteolin glycosides, isoflavonoids (e.g., genistein and tectoridin), and anthocyanins. The coordinated upregulation of structural genes and accumulation of their corresponding metabolites indicates that blue and white light enhance both the core phenylpropanoid pathway and downstream flavonoid branches.

Besides flavonoids, the phenylpropanoid pathway provides precursors for phenolic acids, lignans, and coumarins. Within this branch, 13 DAMs and five DEGs were detected, including caffeic- and ferulic-acid derivatives and lignan intermediates such as pinoresinol-4,4′-O-diglucoside. Most of these compounds showed higher accumulation under blue and white light. Differential expression of COMT family members (*COMT-1*, *COMT-2*, and *COMT-3*) suggests functional divergence in O-methylation steps contributing to lignin- and coumarin-related metabolism.

Together, integrated transcriptomic and metabolomic analyses indicate that red light sustains precursor availability but restricts flux into downstream phenylpropanoid branches, whereas blue and white light promote both gene expression and metabolite accumulation across the phenylpropanoid–flavonoid network, leading to increased production of diverse bioactive compounds in *P. heterophylla* tuberous roots.

### qRT‑PCR validation

**Fig. 8:**
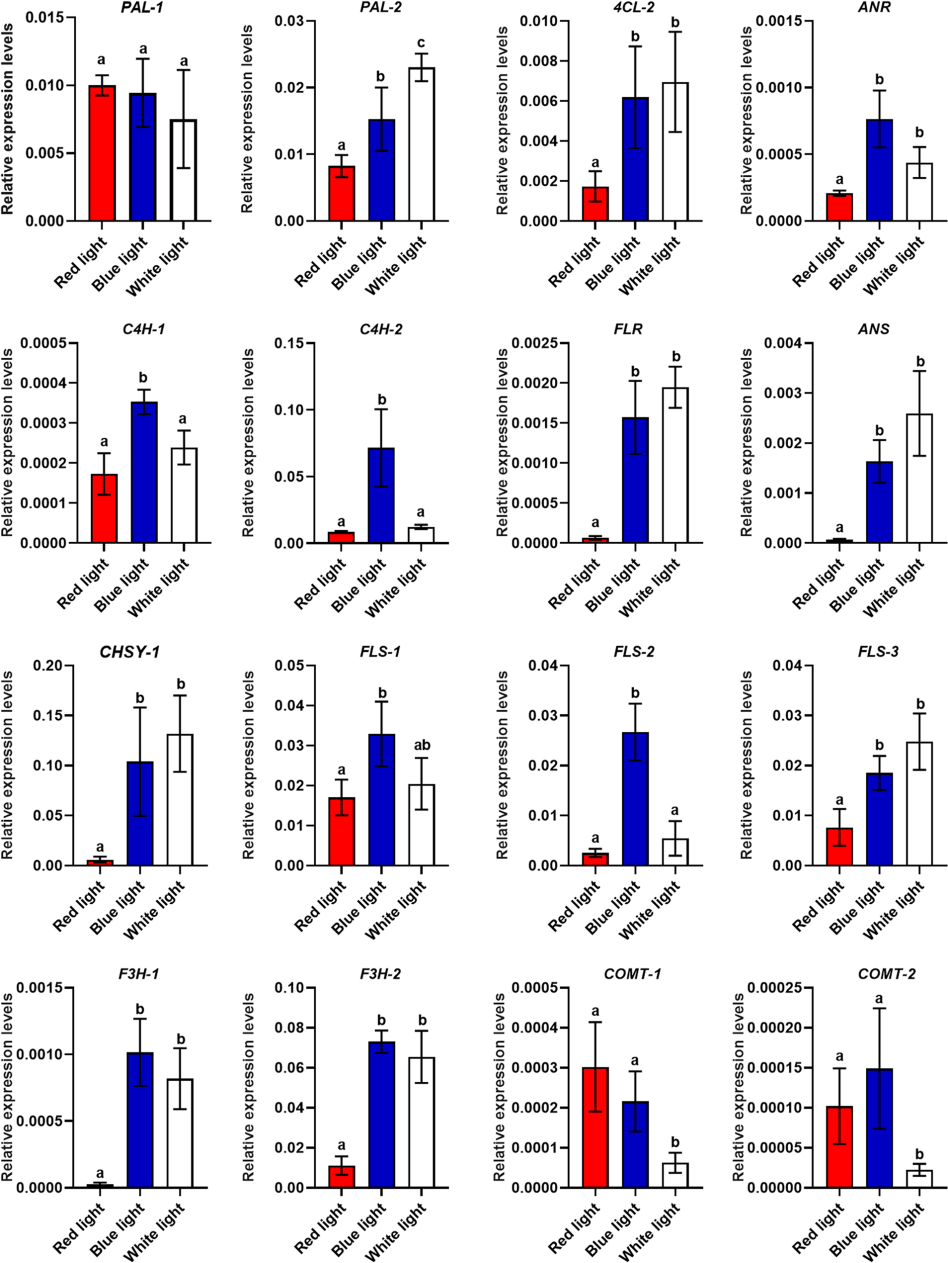
qRT-PCR validation of selected genes under different light-quality treatments. Actin was used as the internal reference gene, with relative expression values being presented as mean ± SD (*n* = 3). The different letters above the error bars represent the significant difference among different groups given by Duncan’s multiple range tests (*P* < 0.05)

To validate the RNA-seq data, qRT-PCR was performed on 16 representative genes involved in phenylpropanoid and flavonoid biosynthesis, including *PAL*, *C4H*, *4CL*, *ANR*, *ANS*, *CHSY*, *F3H*, *FLS*/*FLR*, and *COMT* family members (Fig. 8). qRT-PCR revealed clear light-quality-dependent expression patterns under red, blue, and white light treatments. Notably, several downstream flavonoid biosynthetic genes (e.g., *ANR*, *ANS*, *F3H*, and *FLS* members) showed significantly higher expression under blue and/or white light than under red light. Overall, qRT-PCR results were consistent with RNA-seq trends, while minor discrepancies observed for a few closely related homologs (e.g., COMT members) likely reflect transcript redundancy, alternative isoforms, or differences between de novo unigene assemblies and qRT-PCR primer specificity.

## Discussion

*P. heterophylla* is a traditional medicinal and edible plant widely used in China and is rich in flavonoids, phenolic acids, alkaloids, amino acids, and other bioactive compounds that contribute to its immunomodulatory, antioxidant, and anti-inflammatory activities [15–18]. Although increasing attention has been given to the biosynthesis and regulation of these metabolites, the influence of light quality—a key environmental factor—remains insufficiently understood [2, 3, 9, 27]. In this study, integrated transcriptomic and widely targeted metabolomic analyses were used to compare the effects of red, blue, and white light on the metabolic profiles of P. heterophylla tuberous roots. The results reveal distinct light-quality-dependent metabolic responses and provide novel insights into the light-mediated regulation of specialized metabolism in this medicinal species.

Red light was generally associated with reduced accumulation of flavonoids, phenolic acids, and terpenoids, as reflected by the predominance of downregulated DAMs and decreased levels of compounds such as quercetin and luteolin derivatives. Although several upstream phenylpropanoid genes (*PAL*, *C4H*, and *4CL*) showed moderate induction under red and blue light, overall metabolite accumulation was suppressed under red light, suggesting inhibition of downstream branch flux rather than the entire pathway. KEGG enrichment analysis further supported reduced phenylpropanoid and flavonoid biosynthetic activity under red light. Similar inhibitory effects of red light on secondary metabolism have been reported in *Salvia miltiorrhiza*, *Camellia sinensis*, and *Glycyrrhiza uralensis* [12, 14, 23, 24, 28], likely reflecting a trade-off between primary growth and secondary metabolite production [4, 6, 29]. However, because light responses are often species- and organ-specific, the red-light-mediated suppression observed in *P. heterophylla* requires further physiological validation.

In contrast, blue light markedly promoted specialized metabolism. Flavonoids showed the strongest increase, accompanied by moderate accumulation of alkaloids, amino acid derivatives, and terpenoids. Representative compounds such as luteolin-7-O-glucoside, kaempferol-3-O-glucoside, naringenin-7-O-glucoside, catechin gallate derivatives, and ferulic acid were significantly enriched. These responses were consistent with the strong co-enrichment of phenylpropanoid and flavonoid pathways and with previous reports in *Scutellaria baicalensis*, *Vitis vinifera*, and *Fritillaria cirrhosa* [11, 12, 30–32].

Based on existing models, blue-light photoreceptors such as cryptochromes and phototropins may suppress COP1/SPA activity and stabilize HY5, potentially in cooperation with BBX proteins [5, 7, 8, 33]. In line with this framework, our qPCR results showed that the transcript levels of *COP1* and *SPA* were significantly altered under blue light treatment compared with red and white light, suggesting that components of the COP1/SPA signaling module are responsive to light quality in *P. heterophylla*. Although the downstream regulatory interactions among COP1/SPA, HY5, and BBX proteins were not genetically or biochemically validated in this study, the coordinated upregulation of flavonoid biosynthetic genes, including *CHS*, *CHI*, *F3H*, and *FLS*, under blue light is consistent with a HY5-associated regulatory framework reported in other medicinal plants [9, 11, 22, 34]. Further functional studies will be required to elucidate whether a conserved COP1/SPA–HY5 regulatory module directly operates in *P. heterophylla*. Beyond phenylpropanoid and flavonoid metabolism, light quality also influenced amino acid metabolism. Genes involved in amino acid biosynthesis, including *trpB*, *asnB*, and *TAT*, exhibited consistent expression differences among red, blue, and white light treatments. These transcriptional shifts indicate that light quality modulates primary carbon and nitrogen metabolism, which may indirectly affect precursor availability for secondary metabolite biosynthesis.

Unlike monochromatic red or blue light, white light produced a more balanced metabolic response. White light promoted the accumulation of organic acids, phenolic acids, and terpenoids, with DEGs and DAMs co-enriched in phenylpropanoid, flavonoid, unsaturated fatty acid, and energy-related pathways [3, 21, 22]. This pattern suggests that white light supports coordinated carbon allocation between growth-related and specialized metabolic processes [9, 23, 27]. As a result, white light may serve as a practical baseline spectrum in protected cultivation systems, with red or blue supplementation applied depending on production goals. Because white LEDs vary in spectral composition, responses should be interpreted within the specific spectrum used in this study.

Discrepancies between transcript levels and metabolite accumulation, such as the non-parallel trends between *C3′H* expression and caffeic acid levels, highlight the complexity of metabolic regulation [19, 20, 35]. Caffeic acid is a dynamic intermediate that is rapidly partitioned into downstream pathways, and its steady-state abundance may not directly reflect the expression of a single upstream gene. Such divergence may arise from differences in substrate availability, enzyme activity, metabolic flux, or tissue-specific regulation [29, 36]. Future studies combining gene expression, enzyme activity, and spatial metabolite profiling will be necessary to clarify phenylpropanoid flux regulation in *P. heterophylla*.

Because this study was conducted under controlled tissue-culture conditions, the findings primarily provide mechanistic insight rather than direct agronomic recommendations. Environmental complexity under pot or field conditions may modify spectral responses [9, 13, 14, 37], and validation under production settings is therefore required. Future studies should evaluate composite spectra dominated by blue light but supplemented with red light, while also examining the effects of light intensity and photoperiod on biomass and bioactive compound accumulation [4, 27, 38]. Nevertheless, the results are directly applicable to LED-based in vitro propagation and plant factory systems, where spectral regimes can be precisely controlled to optimize microtuber quality and yield.

From an applied perspective, LED-based spectral management offers a practical approach to improving medicinal quality in *P. heterophylla*. Blue light preferentially enhances flavonoid accumulation, whereas red light may favor biomass production. Optimizing composite spectra that balance these effects could reconcile yield and quality in controlled cultivation systems [3, 6, 29, 38]. By integrating transcriptomic and metabolomic analyses, this study provides a framework linking light quality, carbon allocation, and specialized metabolism and supports the development of rational light strategies for *P. heterophylla* cultivation. Together, these results move beyond the general notion that blue light promotes flavonoids by linking spectrum-dependent carbon allocation with specialized metabolite outputs in a propagation-relevant system through a microtuber-centered multi-omics approach.

## Conclusion

In summary, this study demonstrates that light quality differentially regulates specialized metabolism in *P. heterophylla*. Red light suppressed the accumulation of flavonoids, phenolic acids, and terpenoids but slightly promoted amino acid metabolism. In contrast, blue light strongly promoted flavonoid, coumarin, alkaloid, and terpenoid biosynthesis. White light exerted an integrative effect, supporting balanced activation of both primary and specialized metabolic pathways. Integrated transcriptomic and metabolomic analyses further showed that blue and white light co-activated the phenylpropanoid–flavonoid pathway through the upregulation of key biosynthetic genes. Together, these results provide mechanistic insight into light-driven metabolic regulation and suggest that optimizing LED spectral composition—particularly blue-dominant or red–blue combined spectra—could improve both biomass production and medicinal quality of *P. heterophylla* in tissue-culture nurseries, plant factories, and other protected cultivation systems.

## Supplementary Information

Supplementary Material 1.

Supplementary Material 2.

## Data Availability

The RNA-seq datasets generated and/or analysed during the current study have been deposited in the NCBI Sequence Read Archive (SRA) under the BioProject accession PRJNA1372258. These datasets are currently under embargo and will be made publicly available immediately upon acceptance of this manuscript.
