# Supplementary material for: Integrated transcriptomic and metabolomic analyses reveal light-quality-dependent regulation of specialized metabolism in Pseudostellaria heterophylla
Source: BMC Genomics. 2026 Mar 7;27:377. doi: 10.1186/s12864-026-12717-8 (PMC13081463; doi:10.1186/s12864-026-12717-8)
Supplement: Supplementary file 2 — Supplementary Material 2. [file 12864_2026_12717_MOESM2_ESM.docx]

**Supplemental Information**

Table S1. Sequences of primers used in RT-qPCR assays

| Primer names | sequences |
| --- | --- |
| qPAL-1-F | TGGTGTGCTTTTCGTTTGCC |
| qPAL-1-R | CGGATTCCACCACCGGATAG |
| qPAL-2-F | GCCAATGGAGACACCGAGAA |
| qPAL-2-R | CCTGTCAGCAACTCCGTCTT |
| qC4H-1-F | GAGTCGAGTTTGGGTCCAGG |
| qC4H-1-R | CTTTCTCCAGTGCTCACCGT |
| qC4H-2-F | CGTCGACGCCATATCAGGAA |
| qC4H-2-R | CCGTGCAAAACCGGACATAC |
| q4CL-1-F | GCACAAGAGTCTCGGGACAA |
| q4CL-1-R | ACGCGTAGCGCACATAGTAA |
| qCHSY-1-F | GCTCGATACTACACGTCACG |
| qCHSY-1-R | AGTAGCCTTCCCTTCCTTAGC |
| qFLR-F | GATGTTTGGACCGACCCACT |
| qFLR-R | GCCCATCAGCTAGCTTCCAA |
| qF3H-1-F | AACATTGGCGGGGTTAGAGG |
| qF3H-1-R | CACCGTGATCAACCACCTGA |
| qF3H-2-F | TCTTCACTCAACCGGAGCAC |
| qF3H-2-R | CCTTTTAAGGCCGACAGCAAGA |
| qFLS-1-F | CCTCAAGACCGCCGTAAACT |
| qFLS-1-R | GGGTAGTGGTTCAGTCTCGC |
| qFLS-2-F | GGAGTAGGTTGGGGGTTGTG |
| qFLS-2-R | CCCGCCAAAGGCATTTTGAA |
| qFLS-3-F | TGCAAGGGTATGACACCGAC |
| qFLS-3-R | GTGTTGAGGCCAGATGTGAG |
| qCOMT-1-F | GGGTGCATTTCTCACACCGG |
| qCOMT-1-R | GAATCGACAAACGGGTGACC |
| qCOMT-2-F | CCGAGTCCAAACTGTCTGCT |
| qCOMT-2-R | CATAAAGCCGTTCGGGGCAG |
| qANS-F | ATTATCAATCGCGCTCGGGT |
| qANS-R | GGTTGAGGGCATTTCGGGTA |
| qANR-F | ACGGTACTTCTTTCGCGGAG |
| qANR-R | AGAACCGTCTCCCCTCCTAC |
| Actin-F | CTCCATACCGATAAATGAAGGC |
| Actin-R | CACTGTTCCAATCTATGAGGGTTA |

Table S2. Summary of RNA-seq data quality metrics for *P. heterophylla* samples under different light-quality treatments.

| Sample | Group | Raw Reads | Raw Base(G) | Clean Reads | Clean Base(G) | Error Rate(%) | Q20(%) | Q30(%) | GC Content(%) |
| --- | --- | --- | --- | --- | --- | --- | --- | --- | --- |
| Blue-1 | Blue | 48114302 | 7.22 | 44297532 | 6.64 | 0.01 | 99.5 | 97.39 | 43.9 |
| Blue-2 | Blue | 53665494 | 8.05 | 49593316 | 7.44 | 0.01 | 99.51 | 97.43 | 43.97 |
| Blue-3 | Blue | 50349228 | 7.55 | 46308438 | 6.95 | 0.01 | 99.47 | 97.22 | 43.89 |
| Red-1 | Red | 46502298 | 6.98 | 42313438 | 6.35 | 0.01 | 99.49 | 97.35 | 43.63 |
| Red-2 | Red | 59233012 | 8.88 | 54924490 | 8.24 | 0.01 | 99.47 | 97.25 | 43.84 |
| Red-3 | Red | 49295194 | 7.39 | 45414800 | 6.81 | 0.01 | 99.48 | 97.3 | 43.68 |
| White-1 | White | 66829936 | 10.02 | 60325348 | 9.05 | 0.01 | 99.5 | 97.43 | 44.18 |
| White-2 | White | 49734874 | 7.46 | 46195508 | 6.93 | 0.01 | 99.5 | 97.36 | 44.1 |
| White-3 | White | 44551076 | 6.68 | 41779144 | 6.27 | 0.01 | 99.48 | 97.28 | 44 |

Table S3. Summary statistics of transcriptome assembly results

| Type | Number | Mean Length | N50 | N90 |
| --- | --- | --- | --- | --- |
| Transcript | 142844 | 1379 | 2281 | 616 |
| Unigene | 84963 | 1716 | 2474 | 800 |

Table S4. Summary statistics of functional annotation of genes

| Database | Number of Genes | Percentage (%) |
| --- | --- | --- |
| KEGG | 45517 | 53.57 |
| Nr | 59487 | 70.02 |
| SwissProt | 43754 | 51.50 |
| TrEMBL | 58210 | 68.51 |
| KOG | 37933 | 44.65 |
| GO | 51696 | 60.85 |
| Pfam | 45299 | 53.32 |
| Annotated in at least one Database | 61271 | 72.11 |

Table S6. Abbreviations and full names of enzymes involved in the phenylpropanoid and flavonoid/anthocyanin biosynthetic pathways.

| Enzyme Abbreviation | Full Enzyme Name |
| --- | --- |
| ANR | Anthocyanidin reductase |
| ANS | Anthocyanin synthase |
| C4H | Cinnamic acid 4-hydroxylase |
| CHS | Chalcone synthase |
| CHI | Chalcone isomerase |
| C3H | Coumarate 3-hydroxylase |
| COMT | Caffeate/5-hydroxyferulate 3-O-methyltransferase |
| FLR | Flavone reductase |
| F3’H | Flavonoid 3’-hydroxylase |
| F3H | Flavanone 3-hydroxylase |
| F3′5’H | Flavonoid 3′5’-hydroxylase |
| FLS | Flavonol synthase |
| PAL | Phenylalanine ammonialyase |
| 4CL | 4-Coumarate-CoA ligase |


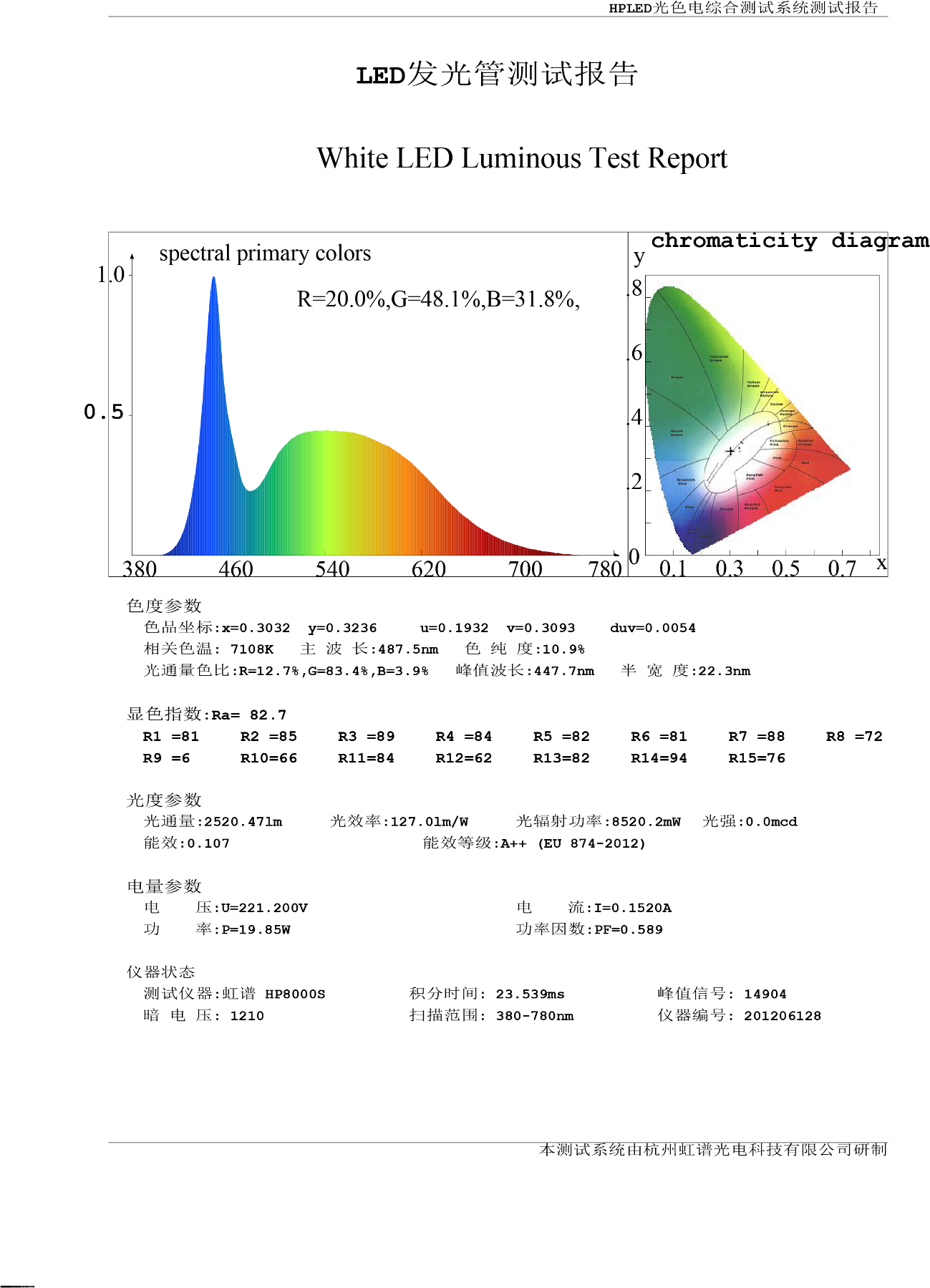


Figure S1. White LED Luminous Test Report


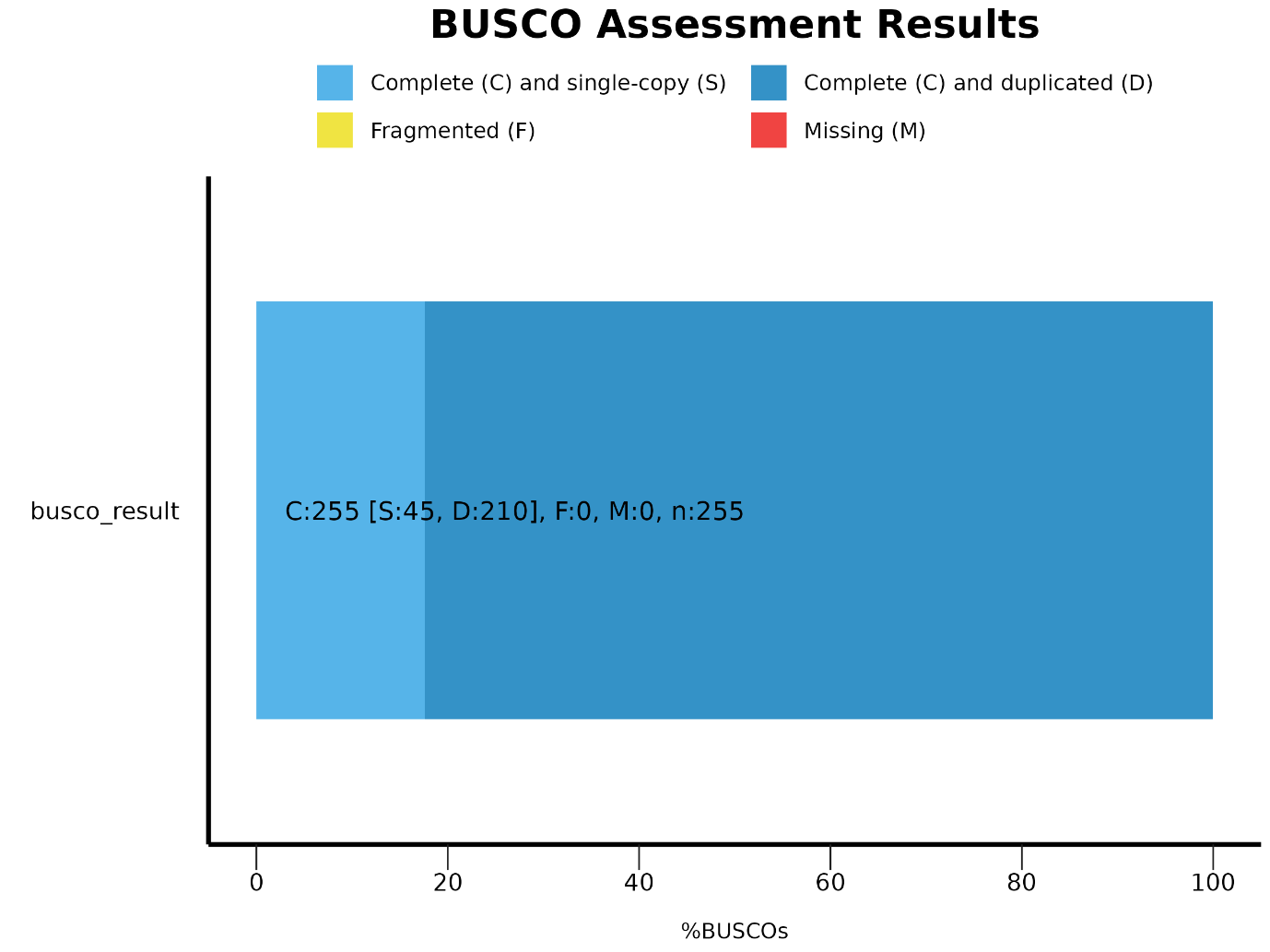


Figure S2. BUSCO assessment of the de novo transcriptome assembly.


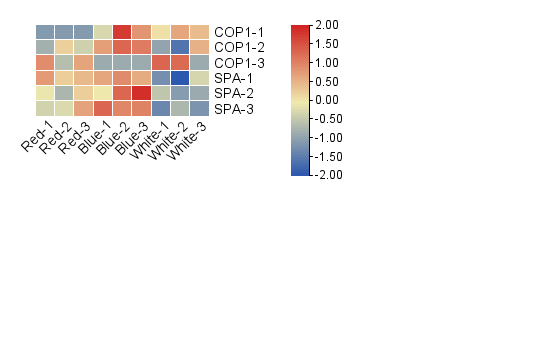


Figure S3. Expression patterns of *COP1*/*SPA* module genes under different light qualities.


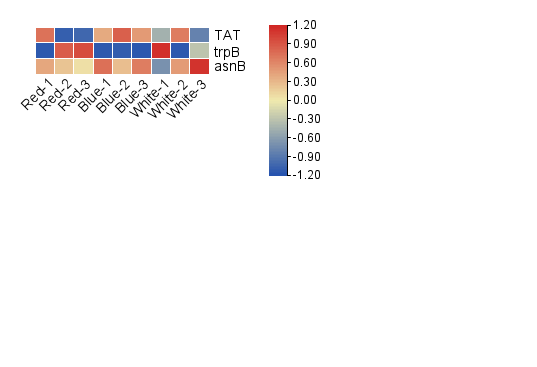


Figure S4. Expression patterns of representative amino acid metabolism–related genes under different light qualities.
